# The Impact of Diagnostic Criteria for Gestational Diabetes Mellitus on Adverse Maternal Outcomes: A Systematic Review and Meta-Analysis

**DOI:** 10.3390/jcm10040666

**Published:** 2021-02-09

**Authors:** Fahimeh Ramezani Tehrani, Marzieh Saei Ghare Naz, Razieh Bidhendi Yarandi, Samira Behboudi-Gandevani

**Affiliations:** 1 Reproductive Endocrinology Research Center, Research Institute for Endocrine Sciences, Shahid Beheshti University of Medical Sciences, Tehran 1985717413, Iran; ramezani@endocrine.ac.ir (F.R.T.); saeigarenaz@gmail.com (M.S.G.N.); razi_bidhendi@yahoo.com (R.B.Y.); 2 Faculty of Nursing and Health Sciences, Nord University, 8049 Bodø, Norway

**Keywords:** adverse maternal outcomes, diagnostic criteria, gestational diabetes, meta-analysis

## Abstract

This systematic review and meta-analysis aimed to examine the impact of different gestational-diabetes (GDM) diagnostic-criteria on the risk of adverse-maternal-outcomes. The search process encompassed PubMed (Medline), Scopus, and Web of Science databases to retrieve original, population-based studies with the universal GDM screening approach, published in English language and with a focus on adverse-maternal-outcomes up to January 2020. According to GDM diagnostic criteria, the studies were classified into seven groups. A total of 49 population-based studies consisting of 1409018 pregnant women with GDM and 7,667,546 non-GDM counterparts were selected for data analysis and knowledge synthesis. Accordingly, the risk of adverse-maternal-outcomes including primary-cesarean, induction of labor, maternal-hemorrhage, and pregnancy-related-hypertension, overall, regardless of GDM diagnostic-criteria and in all diagnostic-criteria subgroups were significantly higher than non-GDM counterparts. However, in meta-regression, the increased risk was not influenced by the GDM diagnostic-classification and the magnitude of the risks among patients, using the IADPSG criteria-classification as the most strict-criteria, was similar to other criteria. In conclusion, a reduction in the diagnostic-threshold increased the prevalence of GDM, but the risk of adverse-maternal-outcome was not different among those women who were diagnosed through more or less intensive strategies. Our review findings can empower health-care-providers to select the most cost-effective approach for the screening of GDM among pregnant women.

## 1. Introduction

Gestational diabetes mellitus (GDM) is one of the most prevalent endocrinopathies during pregnancy and affects 4–12% of all pregnancies depending on the type of diagnostic criteria as well as the prevalence of associated risk factors such as type 2 diabetes (T2DM), body mass index (BMI), advanced maternal age, and ethnicity [1,2,3,4]. Chronic disturbances in maternal β-cell, release of diabetogenic peptides from the placenta, and hormones may play a key role in the pathophysiology of GDM [5]. However, GDM is strongly associated with a higher risk of adverse pregnancy outcomes [6,7], lifelong risk of abnormal glucose tolerance, and diabetes later in life [8,9]. However, appropriate treatment strategies for GDM including lifestyle modifications and pharmacotherapy such as insulin or metformin can significantly decrease related adverse outcomes. In addition, inositol as a nutritional supplementation has been shown to improve glycemic homeostasis during pregnancy and prevent GDM [9,10].

There are ongoing debates regarding the optimum GDM screening strategy. In this respect, the risk of developing postpartum T2DM among women with a history of GDN has been used as the first criteria for the definition of GDM; subsequently, GDM has been defined based on adverse pregnancy outcomes [11] after the Hyperglycemia and Adverse Pregnancy Outcomes’ (HAPO) study, which has shown a linear continuous association between the increasing values of maternal blood glucose and adverse pregnancy outcomes [12]. The International Association of Diabetes in Pregnancy Study Group (IADPSG) [13] and the World Health Organization (WHO) [14] have recommended 75-g oral glucose tolerance test (75 g-OGTT), as the diagnostic criteria for GDM. Although this definition is one of the lowest thresholds for GDM definition, the evidence supporting this endorsement is consensus-based.

Previous reviews have shown associations between GDM and adverse perinatal outcomes just based on the WHO and IADPSG criteria [6] or the IADPSG and Carpenter and Coustan definition [15].

Lack of an evidence-based international definition of GDM may potentially influence the accurate estimation of the risk of adverse maternal outcomes. Therefore, this systematic review and meta-analysis examined the impact of various GDM criteria on the risk of adverse maternal outcomes.

## 2. Materials and Methods

The standard guideline for conducting and reporting meta-analysis [16] was used in this review. The review objectives were as follows:

- To study the pooled risk of adverse maternal outcomes among pregnant women with GDM compared to non-GDM counterparts, regardless of diagnostic criteria;
- To study the pooled risk of adverse maternal outcomes among pregnant women with GDM compared to non-GDM women, according to the various diagnostic criteria;
- To study the association between adverse maternal outcomes and GDM criteria.

### 2.1. Eligibility Criteria

Satisfaction with fulfilling the following criteria was considered for selecting studies: universal screening of GDM; having a population-based design; full description of the GDM screening method and glucose cutoff point in the screening test; reporting the prevalence or risk of short-term maternal outcomes in both GDM and non-GDM groups. Non-original studies and also those with unclear data or insufficient information about the review topic were excluded.

### 2.2. Search Strategy

The authors systematically searched on online databases such as PubMed [including Medline], Scopus, and Web of Science to retrieve original studies published in English on the prevalence, incidence, and risk of adverse maternal outcomes among women with GDM up to January 2020, using the following keywords: (adverse pregnancy outcomes OR pregnancy outcomes OR pregnancy complications OR preeclampsia OR pregnancy-induced hypertension OR gestational hypertension OR PIH OR hemorrhage OR postpartum hemorrhage OR PPH OR placenta abruption OR decolman OR placenta previa OR antepartum hemorrhage OR maternal weight gain OR pregnancy weight gain OR induction of labor OR labor induction OR induced labor OR cesarean sections OR c-section OR abdominal deliveries) AND (pregnancy-induced diabetes OR diabetes in pregnancy OR gestational diabetes mellitus OR gestational diabetes OR GDM).

In addition, the reference lists of the included articles and relevant reviews were manually searched to enhance the possibility of identifying eligible studies.

### 2.3. Study Selection and Data Extraction

Two investigators (M.S.G.N, S.B.G) independently selected manuscripts by the title, abstract, and full text. Next, the following information from each study were extracted: the first author’s name, publication year, study location, sample size, research design, GDM screening characteristics including the screening strategy, details of GDM definition, quality assessment, and outcome measurements in terms of number and prevalence, incidence, or risk of adverse events.

### 2.4. Study Subgroups and Outcomes of Study

The studies were classified into seven sub-groups according to the GDM definition as follows:

(i) IADPSG criteria, one step screening with oral glucose tolerance test (2 h, 75 g GTT); GDM diagnosis: any of the given values are met or exceeded (fasting: 92 mg/dL, BS-1 h: 180 mg/dL, BS-2 h: 153 mg/dL);
(ii) One step screening with 2 h, 75 g OGTT. GDM diagnosis: any of the given valued are met or exceeded (fasting 100 mg/dL, 2 h: 144 mg/dL;
(iii) One step screening with 2 h, 75 g OGTT. GDM diagnosis: any of the given valued are met or exceeded (fasting: 110 mg/dL, 2 h: 140 mg/dL);
(iv) Group 4, one step screening with 2 h, 75 g OGTT. GDM diagnosis: any of the given values are met or exceeded (fasting 100 mg/dL, BS 2 h: 162 mg/dL);
(v) Two step screening with 1 h-50 g Glucose challenge test (1 h-50 g-GCT), values > 140 mg/dL following 100 g OGTT. GDM diagnosis: two values are met or exceeded (fasting: 95 mg/dL, BS-1 h: 180 mg/dL, BS-2 h: 155 mg/dL, BS-3 h: 140 mg/dL or two step screening with 1 h-50 g-GCT, values > 140 mg/dL following 75 g OGTT. GDM diagnosis: two values are met or exceeded (fasting: 95 mg/dL, BS-1 h: 180 mg/dL, BS-2 h: 155 mg/dL, BS-3 h: 140 mg/dL);
(vi) Two step screening with 1 h-50 g-GCT, values > 140 mg/dL following 100 g OGTT. GDM diagnosis: two values are met or exceeded (fasting: 105 mg/dL, BS-1 h: 155 mg/dL, BS-2 h: 165 mg/dL, BS-1 h: 145 mg/dL);
(vii) One step screening with 75 g OGTT. GDM diagnosis: any of the given valued are met or exceeded (fasting: 128 mg/dl, BS2 h: 140 mg/dl).

The adverse maternal outcomes in this review were primary cesarean; gestational weight gain; induction of labor; maternal hemorrhage including antepartum or postpartum hemorrhage, placenta previa, placenta abruption; hypertension-related pregnancy including pregnancy-induced hypertension, preeclampsia, eclampsia.

For quality appraisal, the modified Newcastle-Ottawa Quality Assessment Scale was used [17]. As a validated and standard scale, it assessed nonrandomized studies for inclusion to meta-analyses in terms of the selection of participants, comparability of the study, and assessment of outcomes. Scores above 6, 3–5, and below 3 were interpreted as high, moderate, and low quality, respectively.

The (ROBINS) tool in non-randomized studies of interventions and observational studies was used for assessing the risk of bias [18], which has been recommended by the Cochrane [19]. Five domains of (i) assessment of exposure, (ii) development of outcome of interest in case and controls, (iii) selection of cases, (iv) selection of cases, and (v) control of prognostic variable in cross-sectional studies and 7 domains of (i) selection of exposed and nonexposed cohort, (ii) assessment of exposure, (iii) presence of outcome of interest at the start of the study, (iv) control of prognostic variables, (v) assessment of the presence or absence of prognostic factors, (vi) assessment of outcome, (vii) adequacy of follow up for cohort studies were used for appraisal. The authors classified their judgment on the quality of each study into high risk, unclear risk, or low risk of bias [19].

### 2.5. Statistical Analysis

The Stata version 12 was used for data analysis. Heterogeneity was estimated by I^2^ statistic. The pooled effect size including pooled odds ratio and pooled standardized mean differences of events was calculated using the fixed or random-effects models with Mantel–Haenszel method. Publication bias was evaluated using Begg’s test. The association between the risk of adverse outcome of GDM and its diagnostic criteria as a potential source of heterogeneity was assessed using meta-regression. IADPSG definition criteria were used as the reference group for the comparison. All tests were two-sided and *p* < 0.05 was considered statistically significant.

## 3. Results

### 3.1. Literature Search Results and Quality assessment

**Figure 1:**
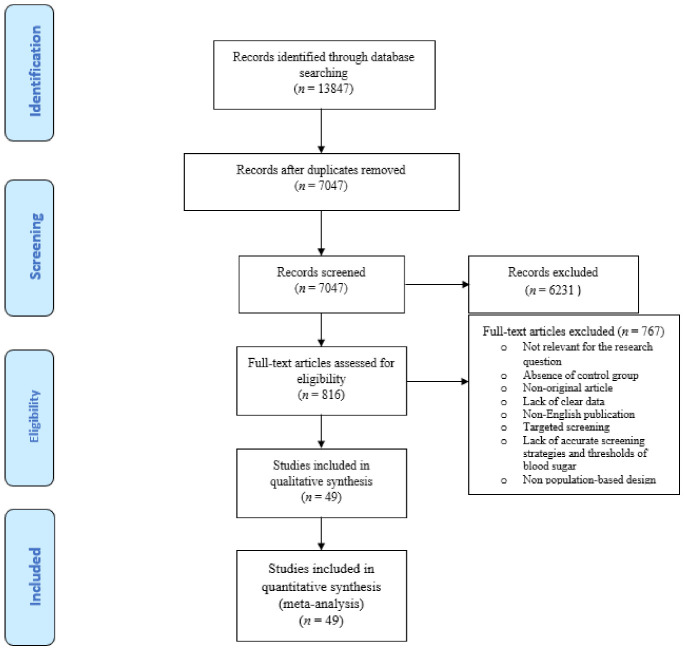
Flow diagram of literature search.

Figure 1 illustrates the flow diagram of the search strategy and study selection.

The search led to 13,847 studies of which 49 studies had the required inclusion criteria and were included in the meta-analysis. The studies’ populations were 1,409,018 pregnant women with GDM and 7,667,546 non-GDM counterparts. Table 1 shows the summary of the studies evaluating the risk of adverse maternal outcomes among GDM and non-GDM populations.

The Supplementary Tables S1 and S2 contain the results of quality assessment. All studies were categorized as high quality [20,21,22,23,24,25,26,27,28,29,30,31,32,33,34,35,36,37,38,39,40,41,42,43,44,45,46,47,48,49,50,51,52,53,54,55,56,57,58,59,60,61,62,63,64,65,66,67,68]. A total of 95.9% studies were prospective or retrospective cohorts [22,23,24,25,26,27,28,29,30,31,32,33,34,35,36,37,38,39,40,41,42,43,44,45,46,47,48,49,50,51,52,53,54,55,56,57,58,59,60,61,62,63,64,65,66,67,68] and 4% were cross-sectional studies [20,21]. In addition, 17 (34.6%) studies used the GDM classification of group 1 [21,22,26,35,38,40,47,48,50,51,53,54,59,60,61,64,67] and IADPSG; 7 (14.2%) group 2 [20,27,28,51,59,65,68], 3 (6.1%) group 3 [32,46,56], 1 (2%) group 4 [51], 19 (38.7%) group 5 [23,24,25,29,31,33,34,36,39,40,42,44,47,53,55,57,58,64,66], 6 (12.2%) group 6 [37,41,43,44,49,52] and 6 (12.2%) group 7 [21,29,30,45,62,63].

It should be noted that 9 studies used more than one GDM classification [21,29,40,44,47,51,53,59,64] as follows: 4 studies used classifications 1 and 5 [40,47,52,63], one used 1 and 2 classifications [59], one used classifications 1, 2 and 4 [51], one used classifications 1 and 7 [21], one used classifications 5 and 6 [44], and finally one used classifications 5 and 7 [29].

In addition, 34.69% of the studies were conducted in the U.S. [22,24,25,31,33,34,36,37,38,41,44,52,53,55,57,58,64], 14.2% in Australia [20,27,28,50,51,65,68], 28.5% in Asia [26,29,32,35,39,40,42,46,47,48,49,60,63,66], and 22.4% in Europe [21,23,30,43,45,54,56,59,61,62,67].

### 3.2. Meta-Analysis and Meta-Regression Results

The overall pooled OR/mean difference (95% CI) of adverse maternal outcomes, its heterogeneity, and the estimation of publication bias among various subgroups of GDM diagnosis criteria, compared to non-GDM counterparts have been presented in Table 2.

**Figure 2:**
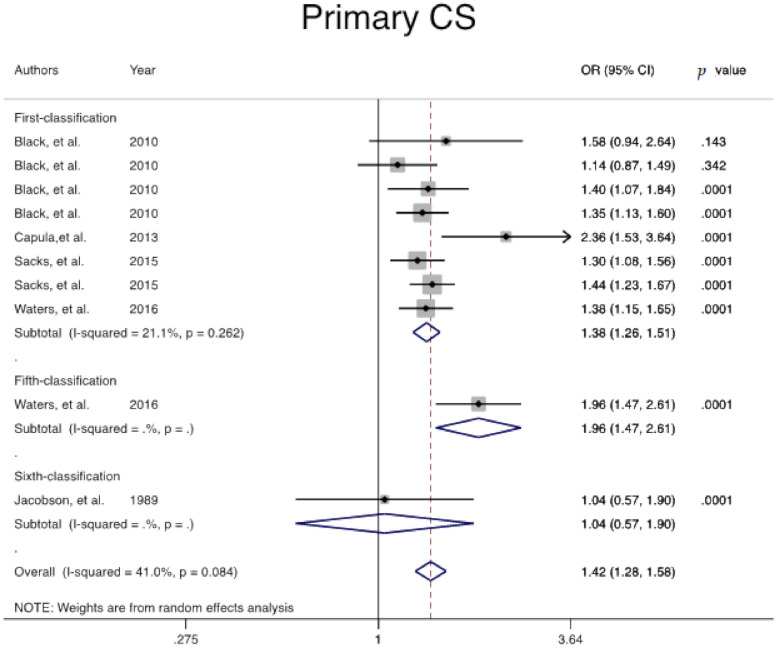
Meta-analysis forest plot of odds ratio (OR) OR for primary cesarean in women with and without Gestational Diabetes Mellitus (GDM) based on different diagnostic criteria.

The odds ratio of primary cesarean among women with GDM, regardless of GDM classification, was 1.4 folds greater than in healthy controls (Pooled overall OR = 1.4, 95% CI: 1.2, 1.5) (Figure 2).

**Figure 3:**
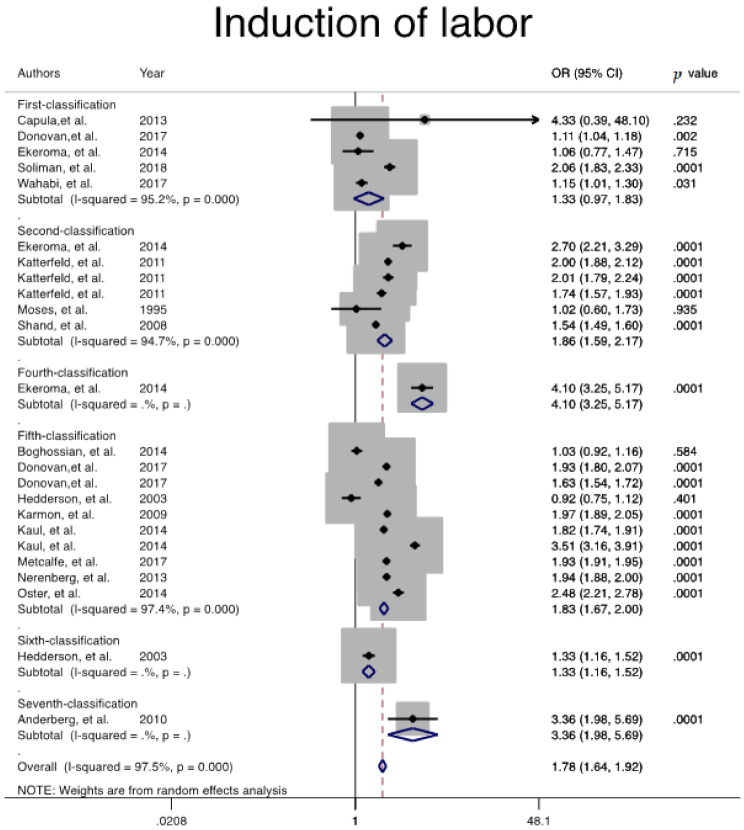
Meta-analysis forest plot of OR for the induction of labor among women with and without GDM based on different diagnostic criteria.

**Figure 4:**
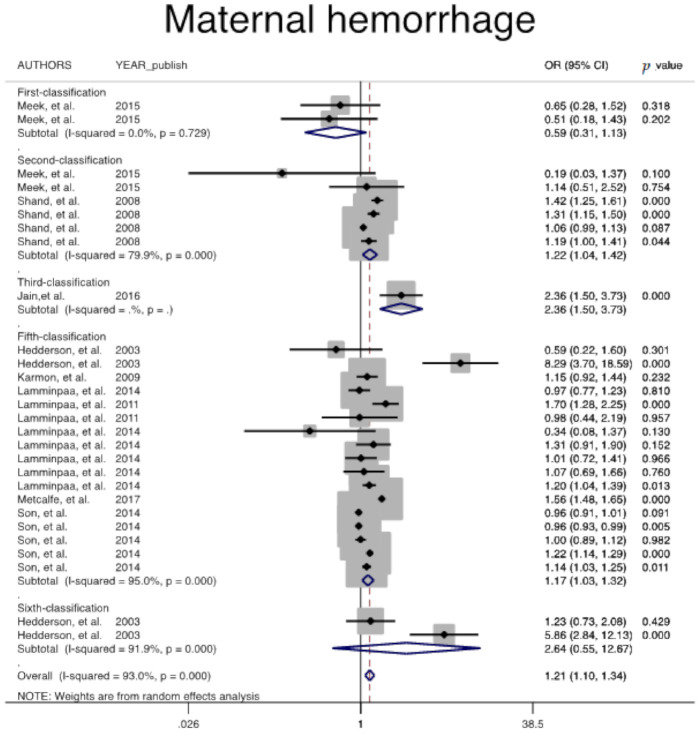
Meta-analysis forest plot of OR for maternal hemorrhage among women with and without GDM based on different diagnostic criteria.

**Figure 5:**
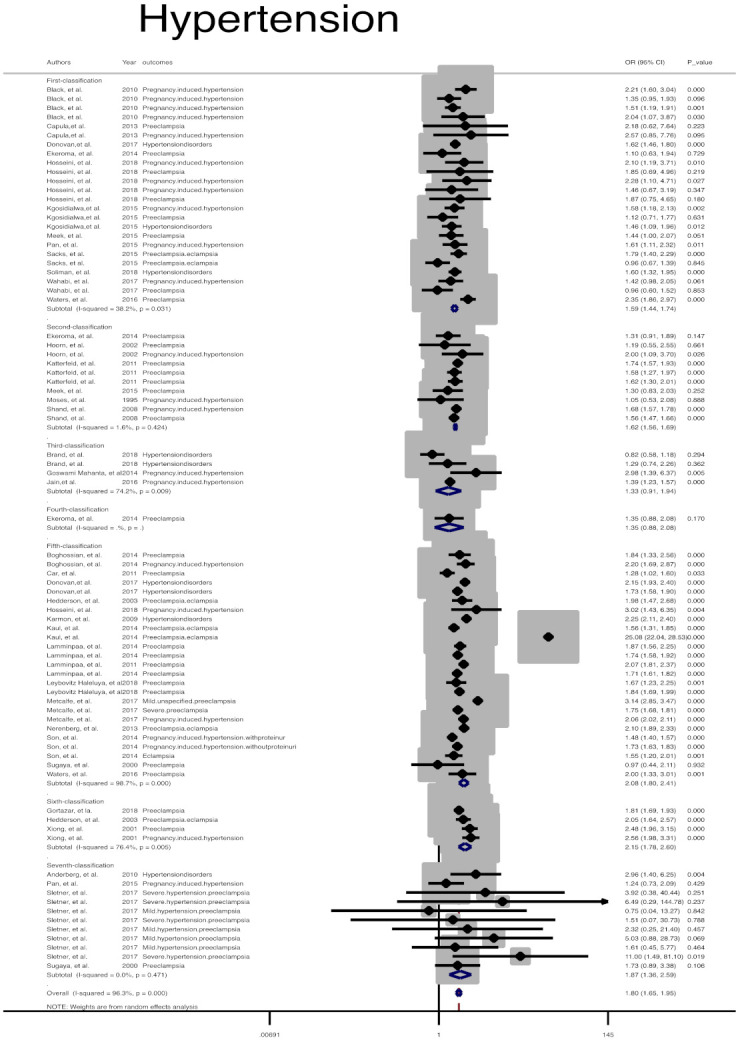
Meta-analysis forest plot of OR for pregnancy-related hypertension among women with and without GDM based on different diagnostic criteria.

In addition, risk of other adverse maternal outcomes, including induction of labor (Pooled overall OR = 1.7, 95% CI: 1.6, 1.9), maternal hemorrhage (Pooled overall OR = 1.2, 95% CI: 1.0, 1.3), and pregnancy-related hypertension (Pooled overall OR = 1.7, 95% CI: 1.6, 1.9) among women with GDM, regardless of GDM diagnostic classification, were significantly higher than non-GDM counterparts (Table 2, Figure 3, Figure 4 and Figure 5).

**Figure 6:**
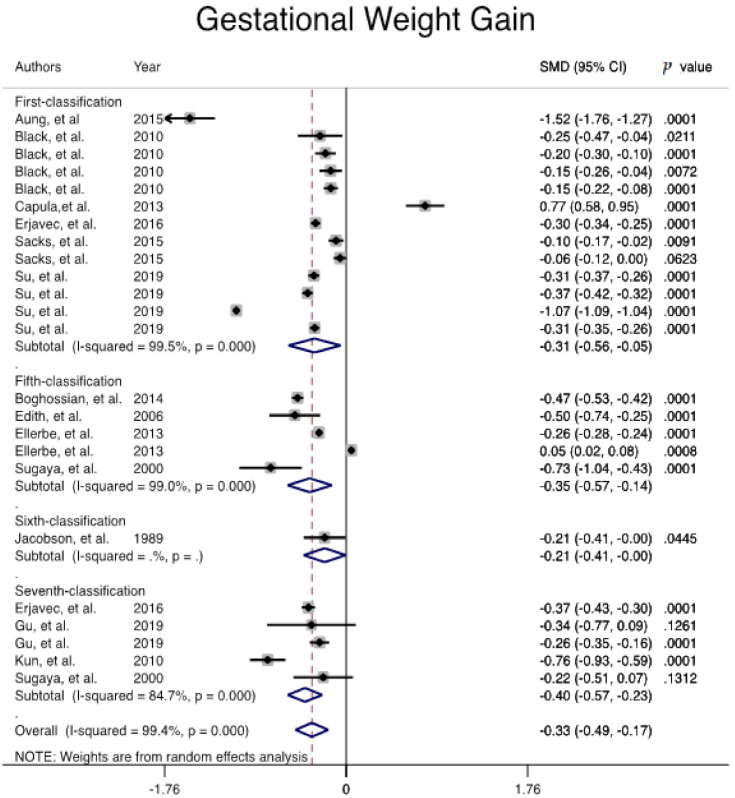
Meta-analysis forest plot of the mean difference of gestational weight gain among women with and without GDM based on different diagnostic criteria.

The gestational weight gain among women with GDM was significantly lower than the non-GDM population, (Pooled overall mean difference = −0.333, 95% CI (−0.492, −0.174) (Figure 6).

Subgroup analysis revealed that the risk of adverse maternal outcomes in women with GDM in all GDM diagnostic classifications were significantly higher than the non-GDM population (Table 2).

**Figure 7:**
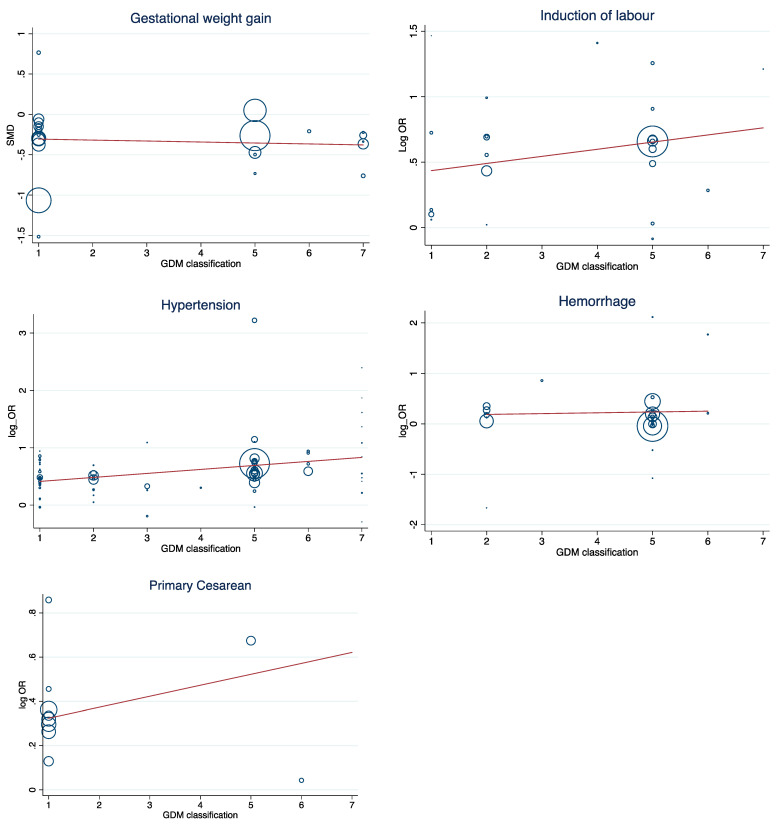
Bubble plot of the meta-regression relationships adverse outcomes and GDM classification.

The results of meta-regression showed that the odds ratio/mean difference were notinfluenced by GDM diagnostic classification. The risk of adverse maternal outcomes in the IADPSG criteria classification, as the strictest criteria, was similar to others (Figure 7).

### 3.3. Results of Publication Bias and Risk of Bias evaluation

According to Begg’s test, no considerable publication bias for various meta-analyses was observed (Table 2). Results of the Risk of Bias evaluation are presented in Supplementary Figures S1A,B and S2A,B. Given that all included studies were observational, the overall risk of bias was low or probably low. However, half of the cross-sectional studies had a probably high risk of bias in the control of prognostic variables. 10% of cohort studies had a probable or high risk of bias in the assessment of exposure and bias in controlling prognostic variables.

## 4. Discussion

Results of this systematic review and meta-analysis demonstrated that GDM, regardless of its diagnostic classification, could increase the risk of adverse maternal outcomes; however, the key finding is that, despite variations in screening approaches, screening methods, and diagnostic threshold values, the increased risk was not influenced by the GDM diagnostic classification.

Despite the wide range of endorsements and guidelines for the diagnosis of GDM in pregnant women recommended by international societies [1,13,69,70,71,72,73,74], there is a strong controversy over the definition of GDM including advice on selective approaches such as universal or risk-based screening, the optimal time for screening in the first and second trimesters, appropriate screening method or criteria for diagnosis, and proper threshold values. Furthermore, there are ongoing debates concerning the risk of adverse pregnancy outcomes and the cost-effectiveness of different screening or diagnostic strategies. However, the aim of almost six decades of research and tremendous efforts has been to reach a global consensus and uniformly accepted guideline with regard to the optimum and cost-effective approach for screening by which the risk of adverse pregnancy outcome is reduced.

The risk of adverse perinatal events using two main GDM diagnostic criteria has been studied by previous reviews. Given that our systematic review and meta-analysis compared all available criteria, it can have a complementary role to the findings of other reviews. For instance, Wendland et al. (2012) [6] in a systematically review and meta-analysis of the relationship between GDM based on the WHO and IADPSG criteria, and adverse events of preeclampsia and cesarean delivery, reported that these criteria could identify women with an elevated risk of adverse perinatal events. The same magnitude for both criteria was reported in our review. Another meta-analysis by Hosseini et al. (2018) [15] assessed the magnitude of the association between GDM using the IADPSG or Carpenter and Coustan criteria and selected adverse perinatal events. They demonstrated that the risk of adverse pregnancy events including preeclampsia, cesarean section, and gestational hypertension increased in both GDM criteria. Although associations with the Carpenter and Coustan criteria were slightly greater, it was not confirmed by the statistical test.

The results of our review demonstrated that despite an increased risk of adverse maternal outcomes among women with GDM, this risk had a similar magnitude for all GDM diagnostic classification. Considering that the use of the strict IADPSG criteria has a significant impact on health care costs and infrastructure capacity with a similar magnitude on short term adverse maternal outcomes, the cost-effectiveness of their use should be defined. Until now, there are not sufficient data to demonstrate the cost-effectiveness superiority of one screening and diagnostic approach over the other [75,76]. In addition, most available cost-effectiveness studies [75,77,78,79,80] were performed in developed societies with higher health economic resources and a lower rate of annual birth than developing and transitional countries [81].

Moreover, the label of GDM, its exhausting treatment, concerns about pregnant women, and unborn health status are some sources of stress, which may lead to a serious psychological problem for some pregnant women and families and could diminish the quality of life [82,83,84]. However, using the optimum cost-effective GDM diagnosis approach with an improved adverse outcome such problems can be prevented.

It is believed that GDM is associated with adverse perinatal events and our meta-analysis confirmed the findings of available literature. Diagnosis of GDM is associated with more pregnancy-related hypertension, and higher rates of induction of labor and primary cesarean section, irrespective of the diagnostic criteria used for GDM. However, insulin resistance has also been hypothesized to contribute to the pathophysiology of adverse outcomes [85]. In our review despite the lower gestational weight gain, an increase in the rate of primary cesarean was seen, which was associated with GDM and an increase in the frequency of induction of labor. It is assumed that gestational weight gain may not be the important factor responsible for the higher odds of cesarean section or induction of labor among women with GDM compared to non-GDM counterparts [7]. Fetal size and macrosomia given fetal insulin response to the elevated glucose level in the body of pregnant women or overtreatment may be associated with an elevated prevalence of cesarean section [7,86]. Moreover, the label of GDM can lead to a tendency toward cesarean section.

Ass the limitations of this review, studies that used the universal screening strategy were selected for inclusion in the meta-analysis. Therefore, studies from north Europe with a low prevalence of GDM that might use a targeted high-risk screening strategy were not included in our review. The short-term maternal outcomes of GDM were considered in our review indicating the need to evaluate the long-term adverse outcomes of GDM based on different diagnostic criteria. Also, given the lack of data on some GDM diagnostic criteria, subgroup analysis for classifications could not be carried out and the lack of a unique definition for each adverse pregnancy outcome may have affected our review findings and their generalizability. Additionally, the effect of diagnostic criteria on outcomes irrespective of GDM treatment strategies might have influenced the results.

## 5. Conclusions

The use of the straighten criteria of the IAPDSG definition can increase the prevalence of GDM among pregnant women. Also, the magnitude of the increased risk of adverse maternal outcomes in all diagnostic criteria was similar. The finding of our review can empower health care providers to select the cost-effective GDM screening approach for pregnant women.

## Figures and Tables

**Table 1:** Characteristics of studies assessing the adverse pregnancy outcome in gestational diabetes mellitus (GDM) and non-GDM population.

Author, Year	Country	GDM Diagnostic Criteria	GDM Characteristics *	Non-GDM Characteristics *	Adverse Maternal Outcome in Women with vs. without GDM, % or Mean (SD)
Capula et al., 2013	Italy	IADPSG	n = 171, Age: 30.8 (3.2), BMI: 22.8 (1.9)	n = 367, Age: 29.3 (3.5), BMI: 21.4 (2.0)	Hypertension: 4.1 vs. 1.6; Preeclampsia: 2.9 vs. 1.4; Labor induction: 1.2 vs. 0.3; gestational weight gain: 10.3 (3.4) vs. 8 (2.8); Primary cesarean section: 29.8 vs. 15.3
Karmon et al., 2009	Israel	CC	n = 10,227	n = 174,029	Hypertensive disorders: 11.6 vs. 5.5 Abruption: 0.8 vs. 0.7; Labor induction: 42.1 vs. 27.0.
Moses et al., 1995	Australia	ADIPS	n = 138, Age: 29.5 (5.3)	n = 144, Age: 28.2 (5.4)	PIH:13.8 vs. 13.2; Labor induction: 26.8 vs. 26.4
Waters et al., 2016	North American	1) IADPSG 2) CC	1) n = 878, Age: 31.0 (5.6), BMI: 31.5 (6.4) 2) n = 261, Age: 32.3 (5.3), BMI: 31.6 (5.8)	n = 5020, Age: 30.1 (5.8), BMI: 28.2 (4.9)	1) Preeclampsia: 14.9 vs. 6.4; Primary cesarean section: 23.9 vs. 17.2 2) Preeclampsia: 14 vs. 6.4; Primary cesarean section: 30.4 vs. 17.2
Gu et al., 2019	China	WHO-1999	GDM with hypertensive disorders of pregnancy: n = 91, Age: 33.8 (3.59), Pre-pregnancy BMI: 25.1 (3.64) GDM without hypertensive disorders of pregnancy: n = 1172, Age: 33.3 (3.49), Pre-pregnancy BMI: 22.9 (3.24)	Non-GDM with hypertensive disorders of pregnancy: n = 261, Age: 32.9 (2.68), Pre-pregnancy BMI: 22.2 (3.04) Non-GDM without hypertensive disorders of pregnancy: n= 261, Age: 32.9 (2.84), Pre-pregnancy BMI: 21.4 (2.96)	Non hypertensive disorder: Gestational weight gain, kg: 16.6 (5.87) vs. 18.2 (6.67) Hypertensive disorder: Gestational weight gain, kg: 19.0 (7.01) vs. 21.3 (6.14)
Shand et al., 2008	Australia	ADIPS	n = 16,727	n = 349,933	Pre-eclampsia: 6.7 vs. 4.4; Gestational hypertension: 6.9 vs. 4.2; Placenta Previa or abruption: 1.6 vs. 1.1; APH: 1.5 vs. 1.1; PPH: 6.3 vs. 6; Severe PPH: 0.9 vs. 0.7; Labor induction: 32.7 vs. 23.9
Anderberg et al., 2010	Sweden	WHO-1999	n = 306, Age: 32 (18–46)	n = 329, Age: 31 (20–42)	Labor induction: 18.6 vs. 6.4
Avalos et al., 2013	Ireland	IADPSG	n = 622, Age: 32.8	n = 4225, Age: 31 (4.9)	GDM without risk factor vs. GDM with risk factor vs. Non-GDM Hypertension: 13 vs. 15 vs. 7
Wahabi et al., 2017	Saudi Arabia	WHO-2013	n = 2354, Age: 31.5 (5.9)	n = 6951, Age: 29.5 (5.7)	Gestational hypertension: 1.8 vs. 1.3; Preeclampsia/superimposed: 1 vs. 1.1; Labor induction: 17.9 vs. 16
Meek et al., 2015	UK	1) IADPSG 2) NICE	1) n = 387, Age: 32.6, BMI: 27.4 2) n = 261, Age: 32.1, BMI: 25.5	n = 2406, Age: 31.4, BMI: 26	1) Pre-eclampsia: 10.1 vs. 7.2; PPH:1 vs. 2; APH: 1.6 vs. 2.4 2) Pre-eclampsia: 9.2 vs. 7.2; PPH:0.4 vs. 2; APH: 2.7 vs. 2.4
Boghossian et al., 2014	USA	ICD-9	n = 1279, Age: 30.3 (4.9); Prepregnancy BMI: 28.9 (7.2)	n = 58,224, Age: 28.1 (4.5), Prepregnancy BMI: 24.9 (5.6)	Gestational hypertension: 4.7 vs. 2.2; Preeclampsia: 3 vs. 1.6; Labor induction: 40.2 vs. 39.4
Kawakita et al., 2017	USA	ICD-9	n = 11,327, Age: 30.8 (6.0), BMI: 34.1 (7.5)	n= 208,355, Age: 27.4 (6.1), BMI: 30.6 (6.1)	Pregnancy-associated hypertension: 11.7 vs. 7.2
Brand et al.2018	UK	Modified WHO-1999	White European: n = 210, Age: 30.2 (5.4), BMI: 28.6 (6.3) South Asian: n = 622, Age: 30.7 (5.3), BMI: 28.2 (5.8)	White European: n = 4537, Age: 26.6 (6.0), BMI: 26.5 (5.9) South Asian: n = 5336, Age: 27.7 (5.0), BMI: 25.2 (5.3)	White European Hypertensive disorders of pregnancy: 6.7 vs. 6.7 South Asian Hypertensive disorders of pregnancy: 5.6 vs. 5.2
Kaul et al., 2014	Canada	CDA-2013	GDM only: n = 7332, Age: 31.9 (5.5) GDM and overweight: n = 1399, Age: 31 (5.2)	n = 213,765, Age: 28.6 (5.6)	GDM only vs. GDM and overweight vs. No GDM, not overweight Pre-eclampsia or eclampsia: 1.9 vs. 5.5 vs. 1.2; Labor induction: 42.1 vs. 58.4 vs. 28.5
Kgosidialwa et al., 2015	Ireland	IADPSG	n = 567, Age: 33.4 (4.9), BMI: 30.5 (6.1)	n = 2499, Age: 31.5 (5.2), BMI: 26.7 (4.8)	Pre-eclampsia: 4.2 vs. 3.8; Hypertensive pregnancy disorders: 11.6 vs. 8.3 PIH: 11.6 vs. 7.7
Donovan et al., 2017	Canada	CDA IADPSG	HAPO 1.75: n = 4308, Age: 31.2 (5.1) HAPO 2–1: n = 5528, Age: 31.6 (5.2) HAPO 2–2: n = 3252, Age: 32.1 (5.2)	Normal 50 g screen: n = 144,191, Age: 28.8 (5.3) Normal 75 g OGTT: n = 21,248, Age: 30.3 (5.3)	Normal 50 g screen: Hypertensive disorders of pregnancy: 5.6; Labor induction: 27.5 Normal 75 g OGTT: Hypertensive disorders of pregnancy: 7.3; Labor induction: 27.7 HAPO 1.75: Hypertensive disorders of pregnancy: 9.1; Labor induction: 29.6 HAPO 2–1: Hypertensive disorders of pregnancy: 9.6; Labor induction: 38.2 HAPO 2–2: Hypertensive disorders of pregnancy: 11.7; Labor induction: 42.3
Kieffer et al., 1999	Michigan	NDDG	n = 19, Age: 29.4 (6.2), BMI: 28.7 (5.7)	n = 353, Age: 24.79 (4.85), BMI: 25.1 (4.21)	Hypertensive disorder: 21.1 vs. 7.16
Ekeroma et al., 2014	New Zealand	1) NZSSD 2) IADPSG 3) ADIPS	1) n = 381, Age: 31.7 (5.5), BMI: 31.8 (10.8) 2) n = 238, Age: 31.4 (5.8), BMI: 32.9 (11.7) 3) n = 608, Age: 31.5 (5.4), BMI: 30.5 (9.8)	n = 1672, Age: 30.0 (5.7), BMI: 30.7 (9.1)	1) Pre-eclampsia: 8 vs. 6 2) Pre-eclampsia: 7 vs. 6 3) Pre-eclampsia: 7 vs. 6
Aung et al., 2015	Cook Islands	Modified IADPSG	n = 94, Age: 36 (28–40), BMI: 34 (30–39)	n = 28 (23–34), Age: 24.79 (4.85), BMI: 31 (26–36)	Pregnancy weight gain (kg): 6 (3–11) vs. 10 (6–14)
Erjavec et al., 2016	Croatia	1) WHO-1999 2) IADPSG	1) n = 953, Age: 30.88 (5.23), BMI: 25.84 (5.28) 2) n = 1829, Age: 31.34 (5.19), BMI: 26.03 (5.64)	1) n = 41,703, Age: 28.77 (5.23), BMI: 23.38 (3.99) 2) n = 37,263, Age: 29.49 (5.33), BMI: 23.38 (4.11)	1) Weight gain: 12.57 (5.62) vs. 14.51 (5.29) 2) Weight gain: 12.50 (5.76) vs. 14.19 (5.71)
Gortazar et al., 2018	Spain	NDDG	n = 35,729, Age: 33.42	n = 704,148, Age: 31.27	Pre-eclampsia: 2.56 vs. 1.44
Zamstein et al., 2018	Israel	ACOG	GDM A1: n = 9460, Age: 32.1 (5.8) GDM A2: n = 724, Age: 33.7 (5.6)	n = 206,013, Age: 28 (5.7)	GDM A1 vs. GDM A2 vs. Non-GDM Hypertensive disorders of pregnancy: 11.2 vs. 18.1 vs. 4.8
Hedderson et al., 2003	California	1) NDDG 2) CC	1) n = 1523 2) n = 840	n = 38,515	1) Pregnancy-induced hypertension: 3.4 vs. 1.9; Preeclampsia or eclampsia:5.8 vs. 2.9; Placenta previa: 0.6 vs. 0.1; Abruptio placentae: 1 vs. 0.8; Labor induction: 18.4 vs. 14.5 2) Pregnancy-induced hypertension: 3.6 vs. 1.9; Preeclampsia or eclampsia: 5.6 vs. 2.9; Placenta previa: 0.8 vs. 0.1; Abruptio placentae: 0.5 vs. 0.8; Labor induction: 13.5 vs. 14.5
Hosseini et al., 2018	Iran	IADPSG	Early-onset GDM: n = 93, Age: 30.7 (4.6), Pre-pregnancy BMI: 26.5 (4.2) Late-onset GDM: n = 78, Age: 31.1 (4.9), Pre-pregnancy BMI: 26.2 (4.7)	n = 758, Age: 28.8 (4.6), Pre-pregnancy BMI: 24.2 (4.1)	Early-onset GDM vs. Late-onset GDM vs. Normal Preeclampsia: 6.5 vs. 6.4 vs. 3.6 Gestational hypertension: 8.6 vs. 12.8 vs. 6.1
Hosseini et al., 2018	Iran	1) IADPSG 2) CC	1) n = 78, Age: 18–45 2) n = 35, Age: 18–45	1) n = 35, Age: 18–45 2) n = 801, Age: 18–45	1) Preeclampsia (OR): 1.5; Gestational hypertension (OR): 1.9 2) Preeclampsia (OR): 2.8; Gestational hypertension (OR): 2.4
Jain et al., 2016	India	DIPSI	N = 8000	n = 7641	PIH: 9 vs. 6; APH/PPH: 0.84 vs. 0.32
Kun et al., 2010	Tolna	WHO-1999	n = 139, Age: 29.6 (5.2), Pregnancy BMI: 25.4 (5.3)	n = 2583, Age: 27.1 (4.9), Pregnancy BMI: 23.1 (4.5)	Weight gain, kg: 9.1 (4.8) vs. 12.9 (5.0)
Leybovitz-Haleluya et al., 2018	Israel	ACOG	GDM A1: n = 9460, Age: 32.1 (5.8) GDM A2: n = 724, Age: 33.7 (5.6)	n = 206,013, Age: 28 (5.7)	GDM A1 vs. GDM A2 vs. No GDM Preeclampsia: 7 vs. 6.4 vs. 3.9
Jacobson et al., 1989	California	NDDG	n = 97, Age: 28.8 (0.5), BMI: 27.6 (0.8)	n = 2107, Age: 26.3 (0.1), BMI: 22.8 (0.1)	Pregnancy-induced hypertension: 3.8 vs. 3.7; Weight gain: 30.2 (1.8) (pounds) vs. 33.0 (0.3)
Pan et al., 2015	China	1) WHO-1999 2) IADPSG	1) n = 257, Age: 29 (2.6), Prepregnancy BMI: 22.9 (3.5) 2) n = 429, Age: 28.8 (2.9), Prepregnancy BMI: 23.9 (4)	n = 16 173, Age: 28.4 (2.8), Prepregnancy BMI: 22.1 (3.3)	1) PIH: 15.8 vs. 4.8 2) PIH: 7.5 vs. 4.8
Son et al., 2014	Korea	ICD-10	n = 78,716, Age: 15–49	n = 1171575, Age: 15–49	Pregnancy-induced hypertension without significant proteinuria: 1.71 vs. 1; Pregnancy-induced hypertension with significant proteinuria: 1.66 vs. 1.13; Eclampsia: 0.08 vs. 0.05; Placenta previa: 1.41 vs. 1.16; Premature separation of placenta: 0.42 vs. 0.42; Postpartum hemorrhage: 7.03 vs. 7.30; Antepartum hemorrhage: 2.29 vs. 2.39
Katterfeld et al., 2011	Australia	ADIPS	Australian born n = 4765 CALD n = 1686 Non-CALD n = 1273	Australian born n = 142,537 CALD n = 23,541 Non-CALD n = 31,814	Australian born Pre-eclampsia: 8.4 vs. 5; Labor induction: 54.3 vs. 37.3 CALD Pre-eclampsia: 5.6 vs. 3.6; Labor induction: 37.6 vs. 25.7 Non-CALD Pre-eclampsia: 7.2 vs. 4.6; Labor induction: 51.9 vs. 35
Sacks et al., 2015	California	IADPSG	1) GDM-1: n = 771, Age: 30.9 (5.6) 2) GDM-2: n = 1121, Age: 31 (5.7)	n = 7943, Age: 26.3 (0.1)	GDM-1 vs. GDM-2 vs. normal Preeclampsia–eclampsia: 4.3 vs. 7.7 vs. 4.4; Primary cesarean delivery: 20.6 vs. 22.3 vs. 16.6
Soliman et al., 2018	Qatar	IADPSG	n = 3027	n = 8995	Hypertensive disorders: 5.5 vs. 3.5; Labor induction: 26.5 vs. 12.4
Xiong et al., 2001	Canada	CDA	n = 2755	n = 8995	Gestational hypertension: 11.4 vs. 4.8; Pre-eclampsia: 1.1 vs. 1.1
Oster et al., 2014	Canada	CDA	n = 1224, Age: 28.8 (6.27)	n = 26,793, Age: 24.7 (5.8)	Pregnancy induced hypertension: 11.3 vs. 4.4; Labor induction: 43.6 vs. 23.8
Sugaya et al., 2000	Japan	1) JSOG 2) WHO-1998	1) n = 55, Age: 29.7 (4.3), BMI: 26.2 (3.4) 2) n = 51, Age: 32.8 (4.3), BMI: 26.5 (4.3)	n = 281, Age: 30 (4.7), BMI: 25.5 (3.3)	1) preeclampsia: 18 vs. 17 2) preeclampsia: 28 vs. 17
Nerenberg et al., 2013	Canada	CDA	n = 15,404, Age: 31.5 (5.4)	n = 407,268, Age: 28.4 (5.6)	Preeclampsia/eclampsia: 2.6 vs. 1.2; Labor induction: 41.9 vs. 27.1
Edith Kieffer et al., 2006	Mexico	ADA-2003	n = 68, Age: 28.6 (0.6), BMI: 25.7 (0.2)	n = 933, Age: 24.8 (0.2), BMI: 28.4 (0.8)	Weight gain (kg): 10.0 (0.6) vs. 13 (0.2)
Goswami Mahanta et al., 2014	India	DIPSI	N = 28	n = 749	Gestational hypertension: 53.6 vs. 28.1
Ellerbe et al., 2013	USA	ICD-9	Non-Hispanic White: n = 8567, Age: 29.6 (5.9), BMI: 29.3(7.3) Non-Hispanic Black n = 4724, Age: 27.5 (6.2), BMI: 31.7 (7.5)	Non-Hispanic White: n = 126,524, Age: 27.0 (5.9), BMI: 25.7 (6.1) Non-Hispanic Black n = 71,939, Age: 24.3 (5.6), BMI: 28.1(7.0)	Non-Hispanic White Gestational weight gain (kg): 11.7 (7.7) vs. 13.7 (7.6). Non-Hispanic Black Gestational weight gain (kg): 11.5 (8.3) vs. 11.1 (8.0)
Sletner et al., 2017	Norway	WHO-1999	Europe Mild: n = 30, Age: 31.2 (29.5), BMI: 25.5 (23.8, 27.2) Moderate/severe: n = 9, Age: 30.6 (27.6, 33.5), BMI: 30.5 (27.4, 33.6) South Asia Mild: n = 9, Age: 30.7 (28.3, 33.0), BMI: 25.3 (23.2, 27.5) Moderate/severe: n = 4724, Age: 30.4 (28.0, 32.7), BMI: 22.7 (20.6, 24.9)	Europe n = 310, Age: 30.6 (30.1, 31.1), BMI: 24.3 (23.8, 24.8) South Asia n = 156, Age: 28.4 (27.7, 29.1), BMI: 23.7 (23.0, 24.3)	Europe Mild vs. Moderate/Severe vs. Non-GDM Mild hypertension/preeclampsia: 10 vs. 0 vs. 7; Severe hypertension/ preeclampsia: 2 vs. 0 vs. 2; inclusion to week 28 GWG: 6.2 (5.2, 7.2) vs. 5.2 (3.4, 7.1) vs. 7.1 (6.8, 7.4), week 28 to birth: 4.0 (2.6, 5.5) vs. 2.0 (-0.4, 4.4) vs. 5.9 (5.5, 6.4) South Asia Mild hypertension/preeclampsia: 7 vs. 14 vs. 3; Severe hypertension/ preeclampsia: 0 vs. 7 vs. 2; inclusion to week 28, GWG: 5.6 (3.9, 7.4) vs. 6.5 (4.7, 8.2) vs. 6.6 (6.0, 7.1), week 28 to birth, GWG: 5.1 (2.9, 7.4) vs. 4.8 (2.5, 7.0) vs. 5.2 (4.5, 5.9)
Zeki et al., 2018	Australia	ADIPS	n = 51135, Age: 32.2 (5.3)	n = 950 678, Age: 29.9 (5.6)	Primary Cesarean: Relative % 13.8 vs. 13.5
Hoorn et al., 2002	Australia	ADIPS	n = 51, Age: 30.9 (5.7), BMI:31.5 (.1)	n = 258, Age: 24.9 (6.3), BMI: 25.5 (5.9)	Gestational hypertension: 45.1 vs. 29.1; Preeclampsia: 19.6 vs. 17.1
Su et al., 2019	China	China National criteria	Underweight n = 1466, BMI: 17.55 (0.79) Normal weight n = 6905, BMI: 20.80 (1.21) Overweight n = 2220, BMI: 23.86 (0.57) Obese n = 2252, BMI: 27.21 (2.15)	Underweight n = 12,336, BMI: 17.54 (0.79) Normal weight n = 36,935, BMI: 20.54 (1.2) Overweight n = 6654, BMI: 23.82 (0.56) Obese n = 4730, BMI: 26.97 (1.97)	Normal weight weight gain, kg: 11.45 (3.98) vs. 13.15 (0.25) Underweight weight gain, kg: 12.53 (3.94) vs. 13.76 (3.93) Overweight weight gain, kg: 10.92 (4.49) vs. 12.29 (4.48) Obese weight gain, kg: 8.87 (4.38) vs. 10.50 (4.35)
Metcalfe et al., 2017	Canada	ICD-10	n = 149,780	n = 2,688,231	Gestational hypertension: 7.93 vs. 4; Mild/unspecified Preeclampsia: 0.32 vs. 0.1; Severe preeclampsia: 2.05 vs. 1.18; Placenta previa: 0.9 vs. 0.58; Labor induction: 35.33 (Rate per 100 deliveries) vs. 22.04
Carr et al., 2011	USA	ICD-9&10	n = 1314, Age: 32.7 (5.7)	One abnormal: n= 1242, Age: 32.3 (5.3) Non abnormal: n= 3620, Age: 32 (5.7)	Preeclampsia (n): 111 vs. 102 vs. 226
Lamminpää et al., 2014	Finland	ICD-10	<35 y: n = 19,422 >35 y: n = 7732	<35 y: n = 210,581 >35 y: n = 45,589.00	<35 y: Normal glucose tol. vs. Diet-treated vs. Insulin-treated Preeclampsia: 4.2 vs. 6.7 vs. 7.7; Placenta previa: 0.2 vs. 0.2 vs. 0.2 Late pregnancy bleeding: 1 vs. 1.2 vs. 1.8 >35 y: Normal glucose tol. vs. Diet-treated vs. Insulin-treated Preeclampsia: 5.1 vs. 8.2 vs. 8.6; Placenta previa: 0.4 vs. 0.5 vs. 0.1; Late pregnancy bleeding: 1.3 vs. 1.3 vs. 1.4
Black et al., 2010	California	IADPSG	single isolated impaired glucose tolerance (i-IGT1) n =391, Age: 32.1 (5.4), BMI: 28.1 (5.6) isolated impaired fasting glucose (i-IFG) n = 886, Age: 30.4 (5.6), BMI: 30.8 (7.1) double-isolated impaired glucose tolerance (i-IGT2) n = 83, Age: 32.3 (5.2), BMI: 27.5 (4.7) IFG + IGT n = 331, Age: 32 (5.1), BMI: 31.8 (7)	n = 7020, Age: 28.6 (5.9), BMI: 26.9 (5.8)	i-IGT1 vs. i-IFG vs. i-IGT2 vs. IFG + IGT vs. No GDM Gestational hypertension: 9.8 vs. 10.8 vs. 13.6 vs. 15.4 vs. 7.2; Primary cesarean section: 12.8 vs. 9.1 vs. 18.1 vs. 8.2 vs. 6.6; gestational weight gain: 119 (30.4) (Ib) vs. 427 (48.2) vs. 23 (27.7) vs. 175 (52.9) vs. 1737 (24.7)

IADPSG: International Association of Diabetes and Pregnancy Study Groups; CC: Carpenter and Coustan; ADIPS: The Australasian Diabetes in Pregnancy Society; WHO: World Health Organization; NICE: The National Institute for Health and Care Excellence; ICD: International Classification of Diseases; CDA: Canadian Diabetes Association; NZSSD: New Zealand Society for the Study of Diabetes; NDDG: National Diabetes Data Group; ACOG: American College of Obstetricians and Gynecologists; DIPSI: Diabetes In Pregnancy Study group India; JSOG: Japan Society of Obstetrics and Gynecology; BMI: Body mass index; CALD: culturally and linguistically diverse. * age and BMI are reported as mean (standard deviation).

**Table 2:** Results of meta-analyses for risk/standardized mean difference adverse maternal outcome among women with gestational diabetes according to different GDM screening strategy group.

Outcomes ^£^	GDM Classification	Sample Size		Heterogenicity		Publication Bias Begg’s Test	Effect Size * (95% CI)	*p*-Value from Meta- Regression
		GDM Group	Non-GDM Group	I^2^ (%)	*p*-Value			
Primary Cesarean	1	4632	49,353	21.1	0.262	0.621	1.3 (1.2, 1.5)	Ref
	Overall	4990	56,480	41	0.084	0.655	1.4 (1.2, 1.5)	--
Induction of labor	1	10,098	183,424	95.2	0.001	0.327	1.3 (0.9, 1.8)	Ref
	2	25,197	549,639	94.7	0.001	0.851	1.8 (1.5, 2.1)	0.144
	5	196,263	4,151,466	97.4	0.001	0.371	1.8 (1.6, 2.0)	0.112
	Overall	233,767	4,925,044	97.5	0.001	0.766	1.7 (1.6, 1.9)	--
Maternal Hemorrhage	2	67,430	1,404,544	79.9	0.001	0.348	1.2 (1.0, 1.4)	^€^ Ref
	5	609,575	9,821,846	95	0.001	0.680	1.1 (1.0, 1.3)	0.867
	6	3046	77,031	91.9	0.001	0.317	2.6 (0.5, 12.6)	0.126
	Overall	688,825	11,315,874	93	0.001	0.523	1.2 (1.0, 1.3)	--
Pregnancy related Hypertension	1	20,021	269,637	38.2	0.031	0.766	1.5 (1.4, 1.7)	Ref
	2	42,287	902,497	1.6	0.424	0.325	1.6 (1.5, 1.6)	0.784
	3	8860	18,263	74.2	0.009	0.497	1.3 (0.9, 1.9)	0.535
	5	771,027	14,009,374	98.7	0.001	0.207	2.0 (1.8, 2.4)	0.38
	6	42,762	959,991	76.4	0.005	0.051	2.1 (1.7, 2.6)	0.160
	7	751	18,674	0	0.471	0.484	1.8 (1.3, 2.5)	0.248
	Overall	886,089	1,618,008	96.3	0.001	0.541	1.7 (1.6, 1.9)	--
Gestational weight gain	1	18,518	142,679	99.5	0.001	0.337	−0.307 (−0.560, −0.054)	Ref
	5	14,689	257,901	90	0.001	0.624	−0.353 (−0.569, −0.137)	0.911
	7	2410	45,271	84.7	0.001	1.000	−0.400 (−0.567, −0.233)	0.988
	Overall	35,714	447,958	99.4	0.001	0.564	−0.333 (−0.492, −0.174)	--

* Effect size represents the odds ratio for all variables, except for weight gain that is the standardized mean difference. ^£^ Analysis was not performed in all subgroups of GDM classifications due to insufficient data. ^€^ As there were not enough studies in the first classification, the second one as a reference group for comparison was used.

## Data Availability

The data presented in the study are available on request from the corresponding author.

## Supplementary Materials

The following are available online at https://www.mdpi.com/2077-0383/10/4/666/s1, Table S1: Quality assessment of studies using the Newcastle–Ottawa Quality Assessment Scale for cohort studies., Table S2: Quality assessment of included studies using the Newcastle–Ottawa Quality Assessment Scale for cross-sectional study, Figure S1: Risk of bias in cross-sectional studies, Figure S2: Risk of bias in cohort studies.
