# Supplementary material for: The Impact of Diagnostic Criteria for Gestational Diabetes Mellitus on Adverse Maternal Outcomes: A Systematic Review and Meta-Analysis
Source: J Clin Med. 2021 Feb 9;10(4):666. doi: 10.3390/jcm10040666 (PMC7916110; doi:10.3390/jcm10040666)
Supplement: Supplementary file 1 [file jcm-10-00666-s001.pdf]

Supplementary material:

Table S1. Quality assessment of studies using the Newcastle–Ottawa Quality Assessment Scale for cohort studies.

	Selection				Comparability	Outcome			
**Author, year**	**Representativeness of the exposed cohort**	**Selection of the non-exposed cohort**	**Ascertainment of exposure**	**No outcome of interest at start of study**	**A: Study controls for age and/or BMI B: Study controls for other confounders**	**A: doctor's diagnosis OR objective measurements B: parent/self-reported doctor's diagnosis OR use of medication**	**Follow-up long enough for outcomes**	**Adequacy of follow up of cohorts**	**Total scores**
Capula et al. 2013	*	*	*	*	**	*	*	*	9*
Karmon et al. 2009	*	*	*	*		*	*	*	7*
Moses et al. 1995	*	*	*	*		*	*	*	7*
Waters et al. 2016	*	*	*	*	**	*	*	*	9*
Gu et al. 2019	*	*		*	**	*	*	*	8*
Anderberg et al. 2010	*	*		*	**	*	*	*	8*
Avalos et al. 2013	*	*	*	*		*	*	*	7*
Wahabi et al. 2017	*	*	*	*	**	*	*	*	9*
Meek et al. 2015	*	*	*	*	**	*	*	*	9*
Boghossian et al. 2014	*	*	*	*	**	*	*	*	9*
Kawakita et al. 2017	*	*	*	*		*	*	*	7*
Brand et al. 2018	*	*	*	*	**	*	*	*	9*
Kaul et al. 2014	*	*	*	*	**	*	*	*	9*

	Selection				Comparability	Outcome			
Author, year	Representativeness of the exposed cohort	Selection of the non-exposed cohort	Ascertainment of exposure	No outcome of interest at start of study	A: Study controls for age and/or BMI<br>B: Study controls for other confounders	A: doctor's diagnosis OR objective measurements<br>B: parent/self-reported doctor's diagnosis OR use of medication	Follow-up long enough for outcomes	Adequacy of follow up of cohorts	Total scores
Kgosidialwa et al. 2015	*	*	*	*		*	*	*	7*
Donovan et al. 2017	*	*	*	*	*	*	*	*	8*
Kieffer et al., 1999	*	*	*	*	*	*	*	*	8*
Ekeroma et al. 2014	*	*	*	*	*	*	*	*	8*
Aung et al. 2015	*		*	*		*	*	*	6*
Gortazar et al. 2018	*	*	*	*	**	*	*	*	9*
Zamstein et al. 2018	*	*	*	*	**	*	*	*	9*
Hedderson et al. 2003	*	*	*	*	*	*	*	*	8*
Hosseini et al. 2018	*	*	*	*	*	*	*	*	8*
Hosseini et al. 2018	*	*	*	*	**	*	*	*	9*
Jain et al. 2016	*	*		*		*	*	*	6*
Kun et al. 2010	*	*	*	*		*	*	*	7*
Leybovitz et al. 2018	*	*	*	*	*	*	*	*	8*
Jacobson et al. 1989	*	*	*	*		*	*	*	7*
Pan et al. 2015	*	*	*	*	*	*	*	*	8*

	Selection				Comparability	Outcome			
**Author, year**	**Representativeness of the exposed cohort**	**Selection of the non-exposed cohort**	**Ascertainment of exposure**	**No outcome of interest at start of study**	**A: Study controls for age and/or BMI B: Study controls for other confounders**	**A: doctor's diagnosis OR objective measurements B: parent/self-reported doctor's diagnosis OR use of medication**	**Follow-up long enough for outcomes**	**Adequacy of follow up of cohorts**	**Total scores**
Son et al. 2014	*	*	*	*	**	*	*	*	9*
Katterfeld et al. 2011	*	*	*	*	**	*	*	*	9*
Sacks et al. 2015	*	*	*	*	**	*	*	*	9*
Oster et al. 2014	*	*		*	*	*	*	*	7*
Soliman et al. 2018	*	*		*		*	*	*	6*
Xiong et al. 2001	*	*	*	*	*	*	*	*	8*
Sugaya et al., 2000	*	*	*	*		*	*	*	7*
Nerenberg et al. 2013	*	*	*	*	**	*	*	*	9*
Edith et al. 2006	*	*	*	*	*	*	*	*	8*
Goswami et al. 2014	*	*		*		*	*	*	6*
Ellerbe et al. 2013	*	*	*	*	*	*	*	*	8*
Sletner et al. 2017	*	*	*	*	*	*	*	*	8*
Zeki et al. 2018	*	*		*	**	*	*	*	8*
Hoorn et al. 2002	*	*	*	*		*	*	*	7*
Su et al. 2019	*	*	*	*	**	*	*	*	9*
Metcalfe et al. 2017	*	*	*	*	*	*	*	*	8*

Author, year	Selection				Comparability	Outcome			Total scores
	Representativeness of the exposed cohort	Selection of the non-exposed cohort	Ascertainment of exposure	No outcome of interest at start of study	A: Study controls for age and/or BMI<br>B: Study controls for other confounders	A: doctor's diagnosis OR objective measurements<br>B: parent/self-reported doctor's diagnosis OR use of medication	Follow-up long enough for outcomes	Adequacy of follow up of cohorts	
Car et al. 2011	*	*	*	*	*	*	*	*	8*
Lamminpää et al. 2014	*	*	*	*	*	*	*	*	8*
Black et al. 2010	*	*	*	*	**	*	*	*	9*

Table **S2.** Quality assessment of included studies using the Newcastle–Ottawa Quality Assessment Scale for cross-sectional study.

Author, year	Selection				Comparability	Outcome		Total scores
	Representativeness of the samples	Sample size	Non-responders	Ascertainment of the exposure (risk factor)	A: study controls for age and/or BMI<br>B: control for any additional factor	Assessment of the outcome<br>a) Independent blind assessment. **<br>b) Record linkage. **<br>c) Self report. *	Statistical test	
Erjavec et al. 2016	*		*	*		**	*	6*
Shand et al. 2008	*		*	*	**	**	*	8*

A:

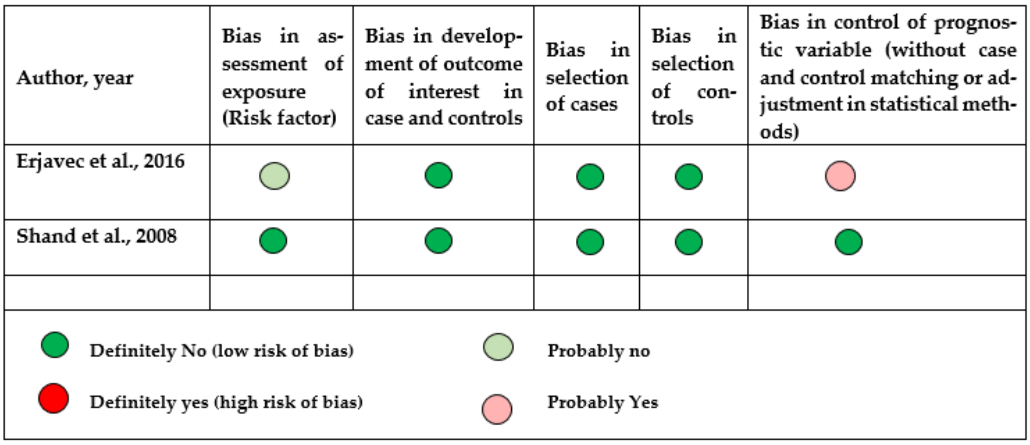

B:.

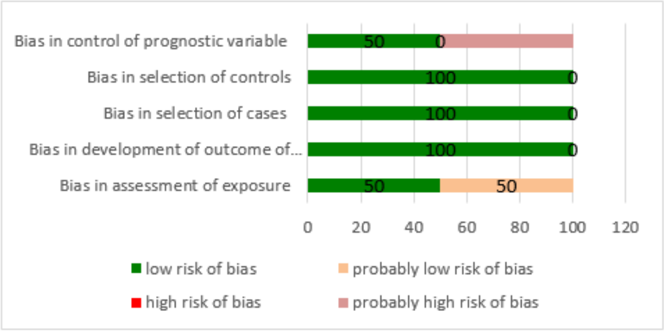

Figure S1. (A and B) Risk of bias in cross-sectional studies.

A:

First Author, year	Bias in selection of exposed and non-exposed cohorts	Bias in assessment of exposure	Bias in present of outcome of interest at start of study	Bias in control of prognostic variables (with matching or adjusting)	Bias in the assessment of the presence or absence of prognostic factors	Bias in the assessment of outcome	Bias in adequacy about follow up of cohorts
Capula et al. 2013	green	green	green	green	green	green	green
Karmon et al., 2009	green	green	green	pink	green	green	green
Moses et al., 1995	green	green	green	pink	green	light green	green
Waters et al., 2016	green	light green	green	green	green	green	green
Gu et al., 2019	green	green	green	green	green	green	green
Anderberg et al., 2010	green	light green	green	green	green	green	green
Avalos et al., 2013	green	green	green	red	green	green	green
Wahabi et al., 2017	green	green	green	green	green	green	green
Meek et al., 2015	green	green	green	green	green	green	green
Boghossian et al., 2014	green	green	green	green	green	green	green
Kawakita et al., 2017	green	light green	green	green	green	green	green
Brand et al.,2018	green	green	green	green	green	green	green
Kaul et al., 2014	green	green	green	green	green	green	green

First Author, year	Bias in selection of exposed and non-exposed cohorts	Bias in assessment of exposure	Bias in present of outcome of interest at start of study	Bias in control of prognostic variables (with matching or adjusting)	Bias in the assessment of the presence or absence of prognostic factors	Bias in the assessment of outcome	Bias in adequacy about follow up of cohorts
Kgosidialwa et al., 2015	green	green	green	pink	green	green	green
Donovan et al., 2017	green	green	green	light green	green	green	green
Kieffer et al., 1999	green	green	green	light green	green	green	green
Ekeroma et al., 2014	green	green	green	light green	green	green	green
Aung et al., 2015	green	pink	green	red	green	green	green
Gortazar et al., 2018	green	green	green	green	green	green	green
Zamstein et al., 2018	green	green	green	green	green	green	green
Hedderson et al., 2003	green	green	green	light green	green	green	green
Hosseini et al., 2018	green	green	green	light green	green	green	green
Hosseini et al., 2018	green	green	green	green	green	green	green
Jain et al., 2016	green	pink	green	pink	green	green	green
Kun et al., 2010	green	green	green	pink	green	green	green
Leybovitz-Haleluya et al., 2018	green	green	green	light green	green	green	green
Jacobson et al., 1989	green	green	green	pink	green	green	green

First Author, year	Bias in selection of exposed and non-exposed cohorts	Bias in assessment of exposure	Bias in present of outcome of interest at start of study	Bias in control of prognostic variables (with matching or adjusting)	Bias in the assessment of the presence or absence of prognostic factors	Bias in the assessment of outcome	Bias in adequacy about follow up of cohorts
Pan et al., 2015	Definitely No	Definitely No	Definitely No	Probably no	Definitely No	Definitely No	Definitely No
Son et al., 2014	Definitely No	Definitely No	Definitely No	Definitely No	Definitely No	Definitely No	Definitely No
Katterfeld et al., 2011	Definitely No	Definitely No	Definitely No	Definitely No	Definitely No	Definitely No	Definitely No
Sacks et al., 2015	Definitely No	Definitely No	Definitely No	Definitely No	Definitely No	Definitely No	Definitely No
Soliman et al., 2018	Definitely No	Probably Yes	Definitely No	Probably Yes	Definitely No	Definitely No	Definitely No
Xiong et al., 2001	Definitely No	Definitely No	Definitely No	Probably no	Definitely No	Definitely No	Definitely No
Oster et al., 2014	Definitely No	Probably Yes	Definitely No	Probably no	Definitely No	Definitely No	Definitely No
Sugaya et al., 2000	Definitely No	Definitely No	Definitely No	Probably Yes	Definitely No	Definitely No	Definitely No
Nerenberg et al., 2013	Definitely No	Definitely No	Definitely No	Definitely No	Definitely No	Definitely No	Definitely No
Edith et al., 2006	Definitely No	Definitely No	Definitely No	Probably no	Definitely No	Definitely No	Definitely No
Goswami Mahantaet al, 2014	Definitely No	Definitely yes	Definitely No	Probably Yes	Definitely No	Definitely No	Definitely No
Ellerbe et al. 2013	Definitely No	Definitely No	Definitely No	Probably no	Definitely No	Definitely No	Definitely No
Sletner et al., 2017	Definitely No	Definitely No	Definitely No	Probably no	Definitely No	Definitely No	Definitely No

First Author, year	Bias in selection of exposed and non-exposed cohorts	Bias in assessment of exposure	Bias in present of outcome of interest at start of study	Bias in control of prognostic variables (with matching or adjusting)	Bias in the assessment of the presence or absence of prognostic factors	Bias in the assessment of outcome	Bias in adequacy about follow up of cohorts
Zeki et al., 2018	Definitely No	Probably Yes	Definitely No	Definitely No	Definitely No	Definitely No	Definitely No
Hoorn et al., 2002	Definitely No	Definitely No	Definitely No	Definitely yes	Definitely No	Definitely No	Definitely No
Su et al., 2019	Definitely No	Definitely No	Definitely No	Definitely No	Definitely No	Definitely No	Definitely No
Metcalfe et al., 2017	Definitely No	Definitely No	Definitely No	Probably no	Definitely No	Definitely No	Definitely No
Car et al., 2011	Definitely No	Definitely No	Definitely No	Probably no	Definitely No	Definitely No	Definitely No
Lamminpää et al., 2014	Definitely No	Definitely No	Definitely No	Probably no	Definitely No	Definitely No	Definitely No
Black et al., 2010	Definitely No	Definitely No	Definitely No	Definitely No	Definitely No	Definitely No	Definitely No

Definitely No (low risk of bias) — Probably no

Definitely yes (high risk of bias) — Probably Yes

B:

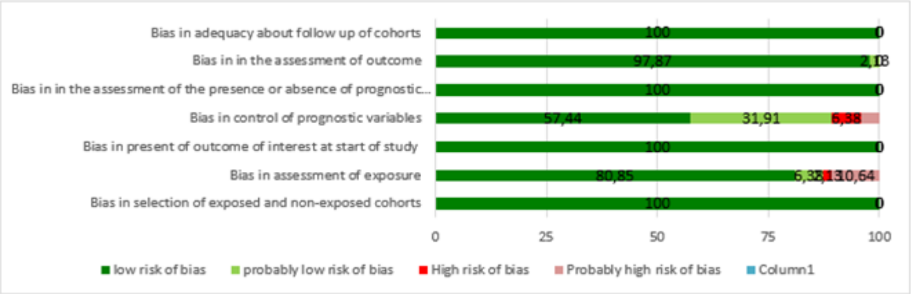

Figure S2. (A and B). Risk of bias in cohort studies.
